# Mucopolysaccharidosis I; Parental beliefs about the impact of disease on the quality of life of their children

**DOI:** 10.1186/s13023-016-0478-z

**Published:** 2016-07-12

**Authors:** A. Soni-Jaiswal, J. Mercer, S. A. Jones, I. A. Bruce, P. Callery

**Affiliations:** Respiratory and Allergy Centre, Institute of Inflammation and Repair, Faculty of Medical and Human Sciences, University of Manchester, Manchester, M13 9PL UK; Willink Unit, Saint Mary’s Hospital, Central Manchester University Hospitals NHS Foundation Trust, Manchester, M13 9WL UK; Paediatric ENT Department, Royal Manchester Children’s Hospital, Central Manchester University Hospitals NHS Foundation Trust, Manchester Academic Health Science Centre, Manchester, M13 9WL UK; School of Nursing, Midwifery and Social work, University of Manchester, Manchester, M13 9PL UK

**Keywords:** Quality of life, Qualitative research, Mucopolysaccharidosis I, Otolaryngology, Obstructive sleep apnoea, Musculoskeletal disease

## Abstract

**Background:**

Hematopoietic stem cell transplants, alongside enzyme replacement therapy and good multi-disciplinary care, have dramatically improved the life expectancy in children with Mucopolysaccharidosis (MPS) I, with better objective and functional outcomes. Despite these improvements, children with both the attenuated (non-Hurler) and severe (Hurler) variants of the disease have marked residual morbidity. Children with MPS I suffer with head and neck disease including obstructive sleep apnoea and hearing loss. The impact of these on quality of life has been poorly researched and no previous work has been published looking at patients’ perception of their own health, an important domain when considering the impact of treatment.

**Methods:**

This exploratory qualitative study aimed to discover the effect of head and neck disease, alongside that of MPS I as a whole, on the quality of life of affected children.

A grounded theory approach was used to conduct this study. Children and their parents were invited to participate in semi-structured interviews. The transcribed interviews were coded and emergent themes explored until saturation occurred.

**Results:**

The families of eleven children with MPS I were interviewed, five with Hurler’s and six with the attenuated non-Hurler’s. Important themes to emerge were- the fear of dying associated with obstructive sleep apnoea, difficulties communicating at school due to the delayed acquisition of language, chronic pain and restricted mobility, physical differences and restricted participation in social activities such as sports secondary to the musculoskeletal disease burden. The overall theme running through the analysis was the desire to fit in with ones peers.

**Conclusion:**

Parents and children with MPS 1 worry about ‘fitting-in’ with broader society. The presence of airway disease has a profound impact on the emotional well being of parents whilst language delay and musculoskeletal disease have the biggest impact on the quality of life of the children themselves. It is important to understand the impact of MPS I on the quality of life of children and their families so that we may improve future treatment and management of this sub-group of children who have an increasing life span.

## Background

Mucopolysaccharidosis type I (MPS I) is an autosomal recessive disorder secondary to a deficiency of the enzyme, α_1_-iduronidase [1–3]. α_1_-iduronidase, is involved in the degradation of the glycosaminoglycan’s (GAG’s) and a deficiency of the enzyme results in a build up of GAG’s within tissues [1]. How accumulation of these GAG’s leads to clinical symptoms is poorly understood [4]. The mutational variability described produces vast clinical phenotypic heterogeneity giving rise to a disease spectrum ranging from the severe (Hurler’s) form, which is characterized by early development of progressive central nervous system (CNS) disease, to the attenuated (Non-Hurler’s) form with CNS sparing [5]. Hurler’s, with its early onset and rapidly progressive symptoms, accounts for seventy percent of children seen with MPS I, with death occurring within the first two decades if left untreated [3, 6]. Although children with Non-Hurler’s have an attenuated form of the disease with slower progression, they still incur significant morbidity [3]. Head and neck disease is frequently seen in children with MPS I, with seventy percent of children suffering multi-level airway obstruction and moderate levels of hearing loss (mean threshold 34 dB) [7, 8].

Hematopoietic stem cell transplantation (HSCT) has become the standard of care for children with Hurler’s; with early transplantation providing improved clinical and functional outcomes, including neurodevelopmental, and a longer life expectancy. The short-term mortality and longer-term morbidity from the HSCT limits its use to children with Hurler’s [9–11], whilst those with non-Hurler’s continue to be managed with enzyme replacement therapy (ERT) [12]. However, with transplant survival rates now in excess of ninety percent, we may see this treatment extended on a case-by-case basis to children with Non-Hurler’s displaying a more severe genotype and phenotype [13]. We have adopted this practice in our unit.

Despite the positive impact of disease modifying treatments on life expectancy, these children are growing older with a substantial burden of residual disease and understanding the challenges that they face in their daily lives is becoming increasingly important. To date, no research has been published, which examines the impact of head and neck disease, or indeed MPS I as a whole, on the quality of life (QOL) of these children or their parents. No previous exploratory work has been done looking at patient’s perception of their own health [14], an important domain when considering the impact of invasive treatments. At present no disease specific tools exist which allow us to assess the impact of MPS I on QOL.

The aim of this qualitative study, using patient interviews, was to explore in-depth the concerns of children with MPS I and their parents, with an emphasis on the impact of head and neck disease on their lives. Through this work, we aim to provide health care professionals with better insight into the patient’s perception of their own health and the priorities that they place on different aspects of their illness. This study also aims to identify important domains for inclusion in an outcome tool designed to measure disease specific impact on quality of life in this sub-group of patients.

## Method

A grounded theory approach was used to obtain an in-depth understanding of the impact of MPS I on the lives of the children and their families. Grounded theory is a qualitative method of narrative research, which allows us to explore the lived-in experience of MPS in greater detail than that offered by subjective questionnaires. In contrast to other narrative methods of research such as phenomology, it can facilitate the generation of theory, rooted in the data, which may help explain why certain aspects of the disease have an impact on QOL [15].

We prospectively recruited eleven patients with MPS I from a large, internationally renowned, tertiary, pediatric metabolic medicine and genetics unit between December 2013 and November 2014.

MPS I represents a heterogeneous group of patients with varying phenotype and severity and therefore purposive sampling was used to include representation of varied forms of the condition. Children were sixteen years or under at the time of interview. National Research and Ethics Committee review was obtained prior to commencement of the study. Informed consent, and assent for those under the age of sixteen, was obtained from all study participants, prior to their participation.

Data was generated using semi-structured interviews. Interviews were conducted when families attended planned appointments or treatments at the unit. An open, conversational style was adopted for the interviews. The researcher sat in a circle of chairs alongside the parents and children, allowing face-to-face conversation. The children were invited to have interviews independently to their parents, allowing their views to be given equal importance. Alternatively they could attend the interviews with the parents if they preferred. Informed consent or assent for those under the age of sixteen was obtained before the interview was conducted. Each interview lasted about an hour and was digitally audio-recorded.

Data collection and analysis were based on the constructivist principles of grounded theory, as described by Charmaz 2006 [16]. We developed a ‘topic guide’ to steer the interviews. The subjects for inclusion were guided by clinical experience because no other qualitative studies exist exploring the lives of families living with MPS I. Topics related to head and neck disease were raised in the interview but rather than specifically privileging these issues, interviewees were encouraged to discuss them within their overall experience of living with MPS. As the interviews were semi-structured and informal, the interviewer asked broad open-ended questions, allowing the interviewees to steer the interview in a direction important for them. The interviewer used probing question to explore responses given by the interviewees.

In keeping with the inductive principles of grounded theory the topic guide developed with time, guided by emerging themes and subjects that appeared to be important to the families. The initial interviews focused on the head and neck disease and the impact it may have on a normal day in the child’s life. Parents were asked to describe ‘a typical day in their child’s life’. However, it soon emerged that parents wanted to discuss the topics ‘education’, ‘language’, ‘mobility’, ‘pain’ and ‘sleep apnoea’ in further detail. Hence, subsequent interviews took on a different format and parents were asked to describe the main impact of their child’s disease on their lives, including education, social integration and future independence. Parents were allowed to determine the direction of the interviews and discuss topics important to them. As the analysis developed, ‘negative’ (i.e. inconsistent) as well as consistent cases were sought and emergent themes were adapted to account for variations, in line with the aim of saturation of categories in grounded theory [16].

A third party transcribed the interviews verbatim. Each transcript was then read to determine emerging themes, guiding further data collection. Field notes were dictated following each interview to make note of the interviewer’s thoughts and impressions. The transcripts were entered and analysed using the Qualitative software NVIVO for Mac^©^ (http://www.qsrinternational.com).

Data analysis was conducted concurrently with data collection. The first stage was open line-by-line coding of the primary data. The emergent codes were grouped into early conceptual categories and through a process of constant comparison grouped further into larger, over-arching categories or themes. Emergent themes were discussed with supervisors as part of a cycle of reviewing the interpretation of data and exploring emergent themes in subsequent interviews. Hence, the identification of categories was iterative, occurring over multiple, progressive cycles of interview-analysis-open coding and reflection. We checked for consistency across cases, searching for negative cases and adapting the categories to account for variation.

This iterative, cyclical, reflective process allowed the theory to be developed inductively from the data. Data were analysed at each stage and further data collection guided by emergent themes. The final data categories were linked in a narrative that accounted for the variations across the sample.

## Results

Over the eleven-month period, nineteen children with MPS I were due to attend the unit for review or treatment on the designated research days and agreed to participate in the study. Eight of them did not attend their designated appointment with the researcher and hence eleven patients were interviewed.

Seven children were female and four were male. Six children (one male and three female) had an attenuated (Non-Hurler) variant of the disease whilst the remaining five children had the more severe (Hurler) form of the disease. The age range interviewed was 6 months to 16 years, mean age 7 years. Two of the children had recently been diagnosed with the illness and were aged 6 months and 18 months respectively. Both of them were female and had Hurler’s syndrome. Both had been started on ERT on diagnosis and were awaiting HSCT at the time of interview. This age range interviewed was important in providing an accurate developmental story covering the journey from initial diagnosis to the age of sixteen, highlighting issues that each group may face.

Within the Hurler group (*n* = 5), the two aforementioned children were awaiting HSCT, two children were post-transplant (5 & 10 years) and one was on long-term ERT (age 16 years). Within the attenuated Non-Hurler (*n* = 6) group, four children were on long-term ERT (12, 12, 15 and 15 years) and two children were post-HSCT (age 18 months and 2.5 years). Both of the transplanted non-Hurlers were diagnosed in the pre-natal period, as they had older affected siblings. Both families opted for their second child to undergo a transplant, rather than be managed on ERT alone.

Three children with Hurler’s were in a special needs educational facility. They all suffered with pronounced developmental delay and varying degrees of neurocognitive disease. Four of the Non-Hurler’s children attended mainstream school, of which two had mild learning difficulties. Four children (2 Hurler and 2 non-Hurler) were under the age of five at the time of interview and did not attend a full-time educational facility.

All children declined the offer to be interviewed separately to their parents. The older children in our cohort participated in the group interviews alongside their parents. The younger children simply observed the interviews.

Pseudonyms have been given to the children to protect their identities.

### Breathing concerns & nocturnal breath holding

A major source of parental concern was the development of obstructive sleep apnoea (OSA) in their children at a very young age. Most of the children had developed it by the age of one year, with some displaying symptoms as early as three to four months old.

Milo’s mother (age 5), *‘At three months old he developed loud snoring, which was audible over the television downstairs. This progressed to breath holding. No one really thought he could be suffering with sleep apnoea at this young age.’*

In many of the children it appears to have been the first cause of concern to parents. This was a fearful time for parents, as many could see that there child was not breathing properly. Some took to sleeping with their child at night so that they could closely monitor them.

Milo’s mother (age 5)*, ‘It used to be a leap of faith putting him to bed. You’d put him to bed and go, god is he going to be there in the morning? It was horrible. I used to sleep with him upright on my shoulder as this stopped him holding his breath as much.’*

Ramona’s mother (age 18 months)*, ‘She wakes up at least every hour and holds her breath while sleeping. I’ve been sleeping close to her since I’ve had her. I’ve tried to put her in a cot, but it just doesn’t work. It stresses her out’.*

The enzyme replacement therapy had a positive impact on the symptoms. Within the first few weeks of being commenced on the ERT treatment, the snoring and breath holding had improved and eventually ceased in most children. Those children with more severe MPS I had undergone an HSCT, which may have contributed to the longevity of improvement.

Milo’s mother (age 5), *‘He had the chemotherapy, followed by the HSCT, and within three months, the severe snoring and breath holding stopped. We are now four years since completion of the transplant and he has not suffered since.’*

The children reported that the disrupted sleep secondary to the OSA made them very tired in the morning, affecting their performance in school.

Mallika’s father (age 15), *‘She used to have symptoms of heavy breathing, to the point where she stopped breathing for a second or two every night. It did not get better with the ERT. A few months ago she had an operation where they removed her tonsils and it has made a huge difference. The symptoms at night have all gone away and she is so much brighter in the mornings.’*

Three children in our cohort were diagnosed with MPS very early (two in the pre-natal period and one at the age of six months prior to the onset of outward symptoms of OSA). All three were started on ERT on diagnosis and then offered a hematopoietic stem cell transplant by the age of six-nine months. To date, these three children remain free of clinical signs of disease (oldest child in this cohort is age three years). However, the improvement in symptoms was not sustained in children with non-Hurlers who had had a late diagnosis (>5 years of age) of MPS. They continued to suffer with progressive disease, requiring a surgery in their early teens, which participants reported led to sustained improvement in their symptoms.

One child in our cohort, Adam (age 16) with Hurler’s has severe multi-level upper airway disease. His upper airway disease and its impact on his life seemed more profound than the other children in our study. His snoring and obstructive sleep apnoea have progressively worsened with increasing age, and at the age of 16, he had been admitted to hospital multiple times requiring oxygen and pressure ventilation support, having to remain in hospital for a week at a time. He also required a continuous positive airway pressure (CPAP) machine at home to support his breathing at night. His parents are now scared that he may completely stop breathing one night and hence vigilantly monitor him whilst at home. They are reluctant to take holidays far from his main hospital, should this happen suddenly. They also worry about him having general aneasthetics. However, they are very accepting of the need for the CPAP machine at home as they feel it improves his quality of life and also his life span.

Thus breathing was a major source of concern from infancy, with improvements in symptoms after commencing ERT and HSCT in all children. However, improvements in breathing were not sustained for children with non-Hurlers who commenced treatment later than five years old.

### The acquisition and development of spoken language

Children with attenuated Non-Hurlers and their parents did not report any concerns with regards to language and hearing. However, parents of children with Hurlers, raised concerns over their children’s delayed acquisition of language, in comparison with their peers and siblings. These children had language delay, with many of them not reaching their early speech milestones, with no discernable language at diagnosis of the illness. Parents felt that this delay was due to a number of factors, including physical changes in their child such as a large tongue, poor hearing or developmental delay due to neurocognitive disease.

Ramona’s mother (age 18 months), ‘*during her newborn hearing screening they could not get a consistent reading and diagnosed her with hearing loss. We didn’t know at the time that she had MPS. She was wearing two hearing aids by three months of age. Despite the hearing aids, she could not say any words at all until the ERT was started four weeks ago. It was difficult for her and she used to be quite clingy, with me having to carry her everywhere. Since the treatment, she is making sounds like ‘dada’ and the other day actually said ‘book’. It was such a special moment.’*

Parents commented that the ERT rapidly improved the speech with children speaking within weeks of it being started, much to the amazement of their parents. Those parents with multiple children commented that although the speech markedly improved with the treatment, it was never as good as the children’s other non-affected siblings or peers at school and the mild speech impediment and delayed grammar, carried through into secondary school. Two parents, with multiple affected children, felt that the children treated with HSCT had better language than the children treated with ERT alone, but was still delayed and lacking in clarity, in comparison with unaffected children.

Milo’s mother (age 5), *‘He was nearly three years old before you could make anything legible out when he spoke. It was predominantly babbling and noises. It has slowly improved and he has progressed to full conversational sentences. However, It is not where a five year old child should be.’*

Mallika’s father (age 15), *‘She still has a delay in her language even at fifteen, she gets stuck with some words and there are others that she simply can’t pronounce. Sometimes she gets embarrassed or cross about this.’*

Some parents felt that the language delay was in part due to their child’s underlying hearing loss. Parents described children having a very positive relationship with their hearing aids. The younger ones consistently asked to wear them whilst the older children felt that they helped them hear everything in their social environment, both at home and at school, allowing them to achieve an education and communicate effectively with others.

Ramona’s mother (age 18 months), *‘She points to them if they are not in. She gets frustrated with me if I don’t put them straight in.’*

Parents described hearing aids providing a vital lifeline to the outside world, especially for the younger children. The importance of hearing for parents was indicated by their expressions of frustration about the apparent low priority given to their audiology appointments by other health care staff, with these being frequently cancelled if they coincided with other hospital appointments.

Ramona’s mother (age 18 months), ‘*She has outgrown her left hearing aid and is completely deaf on that side. We have to rely on just the one working aid. The hearing aid appointment has had to be cancelled twice to make time to come to the hospital for the ERT.’*

Older children got angry when they could not hear in a noisy environment or localize sound effectively. They also got cross when siblings or other children would not repeat things for their benefit. They expressed feeling confused in a noisy or busy environment, as the sound was not clear. Some said their confidence was affected when trying to express themselves or communicate with teachers and peers at school.

In summary, hearing difficulties had important consequences for the lives of children, affecting their ability to communicate and their education, which was a major concern for parents of affected children.

### Musculoskeletal disease, mobility and independence

For many parents and children, pain and stiffness within the muscles and joints, alongside the outward physical appearance of these musculoskeletal manifestations, had a large impact on their children’s quality of life.

Musculoskeletal disease started in infancy, with most children developing curvature of their spine, seen as a lump in the back, or trigger fingers and toes. In many these had led to the diagnosis of the disease. It had also contributed to delayed physical milestones such as crawling and walking and many children were still not mobile into their second year of life. The initiation of weekly enzyme infusions rapidly improved the child’s mobility and energy levels allowing them to catch up with other toddlers their age.

Amanda’s mother (age 12), ‘*within seven days of ERT suddenly she was running around the house…she somersaulted off the back of the sofa. She had never done that in her life ever. She was four and a half.’*

Ramona’s mother (age 18 months), ‘*we started the ERT four weeks ago and she has now started walking. Before we came here she couldn’t crawl or stand up on her own.’*

Neither ERT nor HSCT were reported to stop or reverse the disease process completely and musculoskeletal problems continued to develop as the children grew. In our study even those with the most attenuated form of the disease still had significant disease burden. This had an impact on children’s ability to participate in the most basic activities such as sitting up when laid down, climbing a flight of stairs and walking, perhaps to school. The persistent curvature in their spine stopped them standing straight and they had stiffness in their joints, from their fingers and wrists to their shoulders, neck and lower limbs. This stiffness was also accompanied by pain, brought on by use, in some cases even after ten minutes of walking or writing. Many had developed clawing of their hands.

Ayesha (age 15), *‘I get pain in my legs when I walk to school in the morning. I stop for a few minutes and then start again slowly. Sometimes I lose my balance and fall over.’*

Amanda’s mother (age 15)*, ‘Even now, from a lying position she can’t sit up. I have to help her. The walking is an even bigger concern for us.’*

Children with non-Hurlers were able to attend mainstream school. Climbing up and down stairs was challenging and time consuming and hence for their own safety they left the lesson five minutes earlier than the other children and used the stairs or lift to travel up and down. They had multiple sets of schoolbooks, one at home and one at school to save them carrying a heavy bag. Some of them had a teaching assistant to write for them or to carry their books and bags for them.

Ayesha (age 15), ‘*at school, I have a helper to carry my bag and books between my classes. It means that I don’t get as tired and it stops my shoulders and back from hurting.’*

*Amanda (age 12), ‘I don’t want a classroom assistant. I think it will make me less independent and attract attention. It would be helpful with my writing but I manage by just shaking my hands whilst writing to get rid of the cramp. My friends help with my bag and I take the lift instead of the stairs.’*

Older children, from the age of eight onwards, found it frustrating that they could not participate in sports or run like their friends. Or if they could manage it, they had to adopt a different technique to accommodate the stiffness and the disability.

Andrew (age 12), *‘I play cricket at school but I can’t raise my arm above my shoulder to bowl and so have had to change the way I bowl. I can’t really play football, as you need to be able to run faster than in cricket to get the ball and I can’t manage that. When I play cricket I find the extra weight of the shin pads difficult to carry.’*

Amanda (age 12), *‘I get annoyed at not being able to participate in sports or run. I get tired very easily. I cannot stand properly and walking hurts my back and my feet if it is for longer than ten minutes.*

Children of the same age had also become self-aware and realised that their physical appearance and physical stamina were both different to other children around them. Teenage girls kept a low profile at school and avoided changing in front of their peers in the changing rooms. They felt sad missing out on social activities such as going shopping with friends, as their restricted mobility would mean the need for a wheelchair, causing them further embarrassment.

Amanda (age 12), *‘I don’t want to be different, so I just try to blend in and I don’t tell anybody about my condition.’ ‘I don’t like the way my tummy is swollen and looks so big. Also, the way I stand and walk looks funny. My fingers stick out, people might look at them.’*

This idea of difference from ones peers, despite severe musculoskeletal disability, was not an issue for children who had affected siblings and cousins with MPS I. Perhaps having other children in your immediate surrounding with similar problems normalises the problem, allowing the child to attain a shared identity.

All of the children in our group, including those with an attenuated form of the disease, relied on wheelchairs if they had to walk more than ten-twenty minutes at a single stretch. Parents described children as accepting of the wheelchairs and some felt the children were sometime too easily ready to sit in them and not exerting themselves to their full physical ability. However, Amanda (age 12) did comment that she did not want to use the wheelchair in front of her peers as it made her stand out, highlighting her difference to them. The interviews highlighted that wheelchair use within a mainstream school facility could be difficult and the children preferred to walk around for the day, albeit with assistance or slowly.

Three children with Hurler’s required a special needs education, primarily due to autism or severe learning difficulties. From a musculoskeletal point of view, the big advantage of the specialist schools was that they were on one level and the children could easily use their wheelchairs to get around if necessary. They also offered allied services such as physiotherapy and access to a hydrotherapy pool.

Most parents worried about their child’s future physical health, with some voicing concerns about the children growing into adults who were wheelchair bound, unable to live independently, perhaps becoming institutionalised.

Milo’s mother (age 5), *‘we worry most about his independence, whether he's going to be stuck with his old mum for the rest of his life. I want him, you do for your children, to be educated, independent, working, house, family, just happy, doing the normal, run of the mill things. I wouldn't want to see him in care homes and institutions.’*

It was therefore apparent that children emphasised how mobility problems could highlight their difference to their peers, for example by limiting their involvement in activities. Parents were also concerned about the implications for children’s adult lives.

### The emotional impact of the disease

Participants said that young children, 5–6 years had developed awareness of their condition and their difference to other children around them. As they grew older their awareness increased and girls in particular were conscious of their physical appearance and having less physical stamina than peers. Their parents suggested many had developed shy, introverted personalities when not in their home environment.

Amanda’s mother (age 12), *‘She has a tremendous personality at home but becomes painfully shy when we go out. I’m worried that academically she will be a high achiever but will be an emotional wreck by the time she is in her twenties, burdened down by anxiety and depression.’*

Young people’s wish to minimise their difference was illustrated by a fifteen-year-old girl (Ayesha) who stated during the interview that she did not feel different to her peers and that her physical appearance and severe musculoskeletal disabilities were not a problem for her. However, her mother contradicted this by saying that she was always angry at home, shouting at her mother and siblings and if very frustrated, would resort to deliberate self-harm.

Some parents expressed relief at their child looking outwardly normal and not suffering from intellectual impairment. For them, the mental and physical disabilities that may be linked to the condition were hard to cope with. They expressed the desire for their children to be similar to their peers and fit-in with society at large. One mother said that when she was first informed of her child’s illness, she was shown pictures of children with the severe form of the disease. For her this was the scariest part of the diagnosis and she left the hospital appointment that day feeling horrified. They saw HSCT as an opportunity for their children to live a more normal life.

Maya’s father (age 6 months), ‘*the worst nightmare for a parent is that you have an unhealthy or a disabled child. There is no option for us than to go ahead with the bone marrow transplant. It can give her a normal life.’*

Time off school for treatments and hospital appointments was a concern for parents due to the burden of catching up on missed classes and homework. Many children required extra tuition or quite heavy parental support and encouragement to stop them falling behind at school.

Ayesha’s mother (age 15), *‘Despite the tuition classes, she does not achieve the same grades as her cousins. I am worried that without an education she will not have an independent future after we die.’*

## Discussion

### Summary of findings

Participants highlighted effects on breathing, hearing and mobility when they described living with MPS. These problems were associated with emotional effects and could have major impact on children’s social lives. The presence of multi-level airway disease and secondary obstructive sleep apnoea was a major source of concern for the parents of children with MPS I, with many voicing fears about their child dying as a direct result of this. Parents reported that the obstructive symptoms started as early as three-six months of age, prior to the diagnosis of MPS being established, earlier than the age of two reported in the literature [17, 18]. Seventy-five percent (*n* = 8) of children in our study had suffered with it at some stage of their illness, in keeping with the published literature [19].

Parents of children treated early with ERT and HSCT reported marked and sustained improvement in children’s airway symptoms, with many of them reporting resolution of symptoms after HSCT. Parents of those with non-Hurler’s diagnosed later than 5 years of age, or treated with ERT alone, described minimal improvement in their symptoms, requiring an adenotonsillectomy in adolescence. These reports from a small sample of parental observations are consistent with the findings of a published multi-variant analysis which show that early transplantation in Hurlers and early ERT in non-Hurlers provide the most sustained improvements in airway disease [20].

Participants’ accounts of the effects on their lives appeared related to the severity of MPS I. Those with children with attenuated phenotypes reported limited impact on breathing, hearing or mobility into adolescence whilst those with more severe Hurler’s phenotypes described life limiting airway disease, including both CPAP and oxygen dependence in their late teens. These accounts from a small sample are consistent with the literature which confirms that the severity of the OSA worsens with age, with poorer polysomnography scores seen in post-pubescent patients [21].

The impact on breathing in infancy and early childhood was primarily on the parents, who described becoming stressed and worried about their child’s inability to breathe at night and the fear that they may die. They also described being exhausted from being awake with their child every night and co-sharing their bed at night. Parents described improvements to their stress and sleep following HSCT, which improved children’s symptoms. Breathing problems can also be tiring for children: adolescents with persistent airway obstruction report feeling exhausted in the morning with consequences for their performance at school.

The central theme of the accounts of parents and children alike was that they wanted to achieve social integration and acceptance and ‘fit-in’ with peers. Problems with acquisition of language and mobility were important influences on children’s ability to ‘fit-in’ with peers.

Delayed language appeared to be problematic for all of the children but parents described rapid improvement following ERT treatment. A few children had an underlying hearing loss but despite adequate early rehabilitation of this, parents said that their language acquisition and clarity did not improve until initiation of ERT. Those receiving HSCT in addition to ERT described the greatest improvement. However, participants noticed that a mild degree of language delay and loss of speech clarity persisted even into adolescence.

Participants’ accounts suggest that musculoskeletal disease has a very big impact on the children’s lives. The literature describes that musculoskeletal abnormalities associated with MPS [5, 22], measured using functional outcomes, improves over time with ERT [23]. However, no previous reports of the impact of musculoskeletal disease on the children’s lives have been published. Our study shows that despite ERT, the children continue with a marked burden of musculoskeletal disease, which interferes with the child’s ability to fit in with their peers. This is due to their altered physical appearance, the need for adjuncts such as wheelchairs and being excluded from group activities such as team sports or shopping trips. The physical disability is also responsible for feelings of inadequacy, loss of self-confidence, anger and depression. Parents worry that it may also lead to loss of independence and the inability to seek employment, despite good educational achievement.

With advances in medical therapy, primarily ERT and HSCT, these children are living longer, healthier lives. It is important to understand the impact of the disease on the quality of life of the children and their parents, allowing future treatment decisions to be relevant and appropriate to their specific needs. This exploratory work also highlights important themes, which could contribute to the development of a future patient reported outcome measure that may be used to assess health related quality of life in children with MPS 1.

### Strengths and limitations

To our knowledge, this is the first study of its kind to explore in-depth the perception of parents and children on the impact of head and neck disease on the QOL of children with MPS I and their parents.

This is the first study to report the impact of airway obstruction on children with MPS I and their carers. Extensive research has previously explored the impact of OSA on otherwise healthy children. They show that those with OSA have a significantly poorer quality of life, mainly in the areas of general health perception and emotional parental impact. Other subsets affected are bodily pain, physical functioning and family activities. They also score worse than children with Juvenile Rheumatoid Arthritis (JRA) and asthma, significantly in the subsets parental impact-time and parental-impact emotional, with some impact on behavior and social limitations. Lack of parental sleep and constant worry about the health of the young child may be a source of stress for many parents of children with OSA [24–27]. The broader literature also reveals that fragmented sleep and recurrent hypoxia induced arousals in younger children may cause symptoms of hyperactivity, inattention, poor concentration, disruptive behavior such as fighting, bullying and being quarrelsome, labile emotional behavior and poor school performance [28, 29]. Along with behavioral disturbance, studies have reported cognitive impairment with a reduction in intelligence quotient scores and working memory [30]. Working memory is essential for problem solving and normal psychological development in children. In the older adolescents OSA may cause hyper somnolence in the day, poor concentration and lead to depression [29].

These results are based on parental interviews and provide preliminary data. This would have to be further quantified, in future research studies, using both subjective and objective outcome measures post-transplant, alongside long-term observational studies to see if this improvement is sustained into adulthood. Published clinical trials assessing the benefit of ERT on MPS I report a reduction in the apnea-hypopnea index on serial overnight sleep studies after treatment with ERT is commenced but do not use subjective outcomes measures to quantify the perceived improvement in quality of life [12, 31, 32].

Many important categories have emerged and contributed to our understanding of children and parents’ experience of living with MPS I. Although we drew on the principles and procedures of grounded theory methodology, we have identified insights into experience rather than developed a full-grounded theory. Although the children were participants in the interviews, the principal data source is parents’ accounts and we must be aware that parents and children’s accounts of living with chronic diseases can differ, particularly as they relate to emotional and social well being [33].

## Conclusion

Parents and children with MPS 1 worry about ‘fitting-in’ with broader society and their peers. The presence of airway disease in the children has a profound impact on the emotional well being of parents whilst language delay and musculoskeletal disease have the biggest impact on the quality of life of the children themselves. It is important to understand the impact of MPS I on the quality of life of children and their families so that we may improve future treatment and management of this sub-group of children with an increasing life span.

## Abbreviations

CNS, Central nervous system; CPAP, Continuous positive airway pressure; ERT, Enzyme replacement therapy; GAG’s, Glycosaminoglycan’s; HSCT, Hematopoietic stem cell transplantation; JRA, Juvenile Rheumatoid Arthritis; MPS I, Mucopolysaccharidosis type I; OSA, Obstructive sleep apnoea; QOL, Quality of life
